# The Vicious Cycle of Inflammation: How Obesity, Dialysis Catheters, and NETosis Determine Albumin Levels and Prognosis in Hemodialysis Patients

**DOI:** 10.3390/ijms27125591

**Published:** 2026-06-20

**Authors:** Julia Lecyk, Martyna Lica-Miler, Alicja Kwiatkowska, Izabela Szubert, Violetta Dziedziejko, Zuzanna Marcinowska, Patrycja Kapczuk, Krzysztof Safranow, Ewa Kwiatkowska

**Affiliations:** 1 Clinical Department of Nephrology, Transplantology and Internal Medicine, Pomeranian Medical University, 70-111 Szczecin, Poland; 69898@student.pum.edu.pl (J.L.); 67851@student.pum.edu.pl (M.L.-M.); 68771@student.pum.edu.pl (A.K.); 61411@student.pum.edu.pl (I.S.); 2 Department of Biochemistry and Medical Chemistry, Pomeranian Medical University, 70-111 Szczecin, Poland; violetta.dziedziejko@pum.edu.pl (V.D.); zuzanna.marcinowska@pum.edu.pl (Z.M.); patrycja.kapczuk@pum.edu.pl (P.K.); krzysztof.safranow@pum.edu.pl (K.S.)

**Keywords:** NET, neutrophile extracellular traps, hemodialysis, inflammation, body composition, mortality

## Abstract

In hemodialysis patients, Body Mass Index is insufficient in assessing their nutritional status due to the ‘obesity paradox’ and the association between body composition and inflammation. This study assessed the relationship between body composition, traditional inflammatory markers, the new NETosis indicators (neutrophil extracellular traps), and their association with 12-month mortality. The study included 99 maintenance hemodialysis (HD) patients. Their body composition was assessed using bioelectrical impedance analysis. Blood serum was tested for inflammatory markers (hs-CRP-high sensitive c-reactive protein, IL-6 interleukin-6, TNF-α tumor necrosis factor alfa, IL-1β interleukin-1 beta), NETosis markers (citrullinated histone CH3, myeloperoxidase -MPO, elastase), and nutritional status parameters (albumin, transferrin). No correlation between BMI -body mas index and inflammation was demonstrated. Higher adipose tissue, particularly visceral, was significantly positively correlated with IL-6 and hs-CRP levels, while muscle mass was negatively correlated with inflammation. Dialysis catheter use was associated with higher CH3 levels (NETosis indicator) and lower albumin concentrations. Low albumin levels and high TNF-α levels were independent predictors of death. Body composition, rather than BMI, is associated with the severity of inflammation. Visceral obesity is positively correlated with increased inflammation, while muscle mass shows an inverse association. Dialysis catheters are linked to higher NETosis markers and lower albumin levels, which are associated with a poorer prognosis.

## 1. Introduction

Body Mass Index in HD patients is a complex issue and its interpretations differ from those for the general population. Traditionally, BMI has been used as a tool to assess underweight, normal weight, overweight, and obesity. However, in HD patients, BMI often does not accurately reflect the actual body composition and can be misleading when assessing the patient’s nutritional status and mortality risk. The obesity paradox observed in HD patients means that a higher BMI—even within the overweight and obese ranges—is often associated with better survival compared to patients with a low or normal BMI [1,2,3]. There are several possible explanations for this phenomenon. A higher BMI may indicate greater energy reserves, which is beneficial in the face of frequent illnesses and catabolic states typical of kidney failure patients [4]. In addition, a higher BMI may indirectly reflect greater muscle mass, which is also associated with better outcomes in these patients. It is worth noting, however, that a high BMI does not always indicate a better nutritional status; rather, it may simply point to greater adipose tissue reserves, which are not necessarily a beneficial circumstance.

Interpreting BMI in dialysis patients requires caution and consideration of additional factors. The BMI value alone does not take into account body composition, including muscle mass and body fat. Crucially, the relationship between altered body composition and chronic inflammation in ESRD (end stage renal disease) is profoundly bidirectional. While expanded adipose tissue—particularly visceral fat—functions as an active endocrine organ that directly generates and propagates inflammation through the hypersecretion of pro-inflammatory adipokines and cytokines, such as interleukin-6 and tumor necrosis factor-alpha [5,6,7], systemic chronic inflammation acts as a potent driver of muscle wasting and suppresses visceral protein synthesis [8,9]. This phenomenon is referred to as the malnutrition, inflammation, and atherosclerosis (MIA) syndrome [10]. It is characterized by the coexistence of malnutrition, chronic inflammation, and accelerated atherosclerosis. For this reason, in addition to BMI, other parameters should be taken into account, such as waist circumference, muscle mass assessed through various methods (e.g., bioelectrical impedance, DEXA), as well as markers of nutritional status (e.g., albumin, prealbumin, transferrin) and inflammatory markers. The aim of our study was to assess the relationship between body composition, inflammation, and nutrition.

In addition to the commonly used markers of inflammation, we also assessed NETosis (Neutrophil Extracellular Traps formation). Neutrophil extracellular trap (NET) formation—a regulated process termed NETosis, wherein activated neutrophils release extracellular webs of DNA, histones, and granular enzymes like myeloperoxidase (MPO) and elastase—has emerged as a major mediator of endothelial injury and chronic microinflammation [11,12,13]. In HD patients, neutrophils are kept in a state of constant, multifaceted mobilization triggered by bio-incompatible dialyzer membranes, vascular access devices, and the intradialytic influx of high bicarbonate concentrations [14,15,16]. Excessive or dysregulated NETosis directly promotes vascular damage and accelerates the progression of the MIA syndrome [12,17]. Importantly, a landmark study first documented the critical clinical impact of this pathway in 2017, demonstrating a direct correlation between elevated baseline NET activity and overall mortality within the HD population [18]. Despite its clinical relevance, the precise interplay between ongoing NETosis, specific body tissue compartments, and visceral protein synthesis remains poorly characterized.

To address these gaps, the aim of our study was to systematically evaluate the relationships between bioimpedance-derived body composition metrics, classical systemic inflammatory markers, and novel indicators of NETosis activity, while determining their collective association with 12-month all-cause and cardiovascular mortality in maintenance hemodialysis patients.

## 2. Results

Table 1 presents the characteristics of the study group.

### 2.1. Nutritional Status

#### 2.1.1. BMI

The four groups according to BMI are presented in Table 2.

#### 2.1.2. Nutritional Status Groups According to Body Composition

Table 3 presents the nutritional status groups according to body composition.

#### 2.1.3. Markers of Nutritional Status

Table 4 presents the distribution of nutritional status markers (albumin and transferrin) among patients, stratified by levels within or below the reference laboratory range.

### 2.2. Correlations

**Figure 1:**
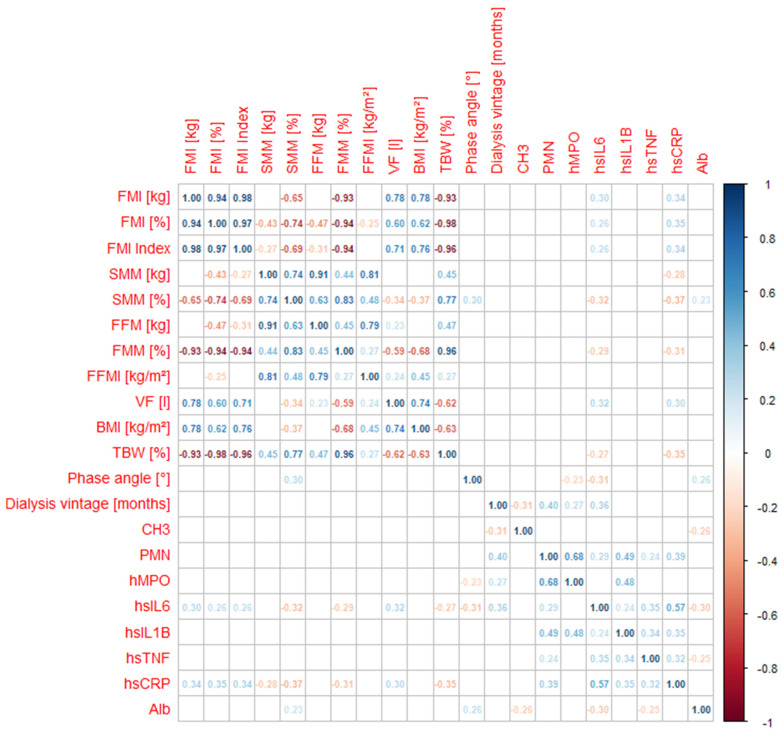
Statistically significant Spearman’s rank correlation coefficients. Abbreviations: FMI [kg]—fat mass; FMI [%]—fat mass percentage; FMI—fat mass index (kg/m^2^); SMM [kg]—skeletal muscle mass; SMM [%]—skeletal muscle mass percentage; FMM [kg]—fat free mass; FMM [%]—fat free mass percentage; FFMI—fat free mas index (kg/m^2^); VF [l]—visceral vat; BMI—body mass index (kg/m^2^); TBW—total body water (%); CH3—citrunillated histone; PNM—polymorphonuclear elastase; hMPO—myeloperoxidase; hs IL-6—high-sensitivity interleukin 6; hsIL1B—high-sensitivity interleukin 1 beta; hs TNF—high-sensitivity tumor necrosis factor; hsCRP—high-sensitivity Creactive protein; Alb—albumin.

Figure 1 presents the statistically significant Spearman’s rank correlation coefficients.

The correlogram figure shows pairwise Spearman correlations between body composition indices (FFMI, FMI, VAT, SMM%) and inflammatory and NET markers (IL-6, TNF-α, hs-CRP, CH3, MPO, PMN). The strongest positive correlations were observed between VAT and IL-6 (rho = 0.0.32, *p* < 0.05) and between FMI and hs-CRP (rho = 0.35, *p* < 0.05). SMM% correlated inversely with IL-6 and hs-CRP (rho = −0.32 and −0.37, respectively; *p* < 0.05). NET markers correlated with dialysis vintage and with catheter presence (see text and Table 5).

### 2.3. Comparative Analysis

**Figure 2:**
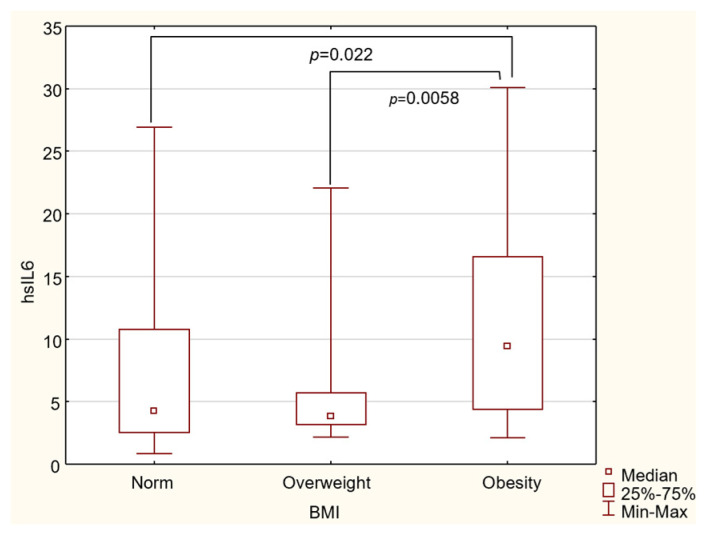
Comparison of hsIL-6 in normal BMI groups with those with overweight and obesity.

Patients with a normal BMI were compared with those who were overweight or obese, and higher IL-6 levels were found in the overweight and obese group (*p* = 0.02 and *p* = 0.0058, respectively) (Figure 2).

**Figure 3:**
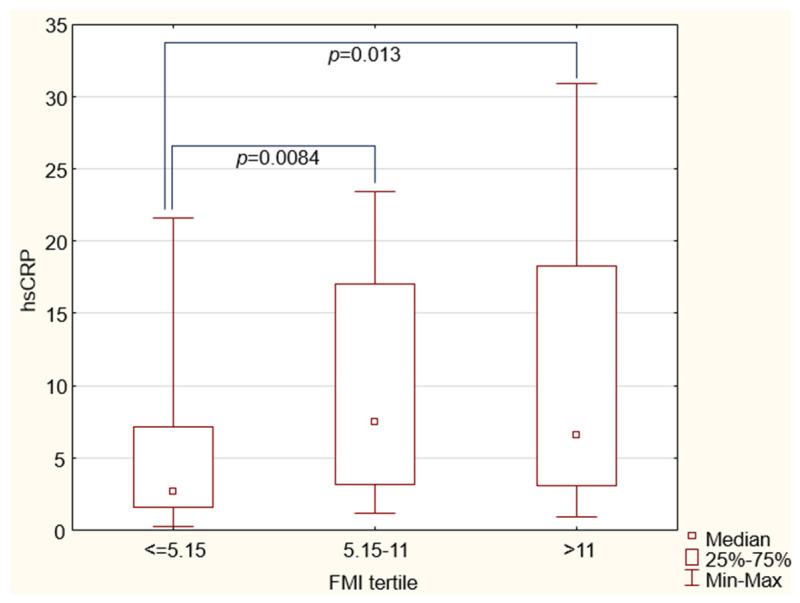
Comparison of hsCRP levels stratified by FMI tertiles (Low, Medium, High).

Fat Mass Index (FMI) proved to be a significant differentiator for systemic inflammation. Specifically, hs-CRP concentrations were significantly higher in both the medium and high FMI tertiles compared to the low FMI tertile (*p* = 0.008 and *p* = 0.01, respectively). Figure 3. 

**Figure 4:**
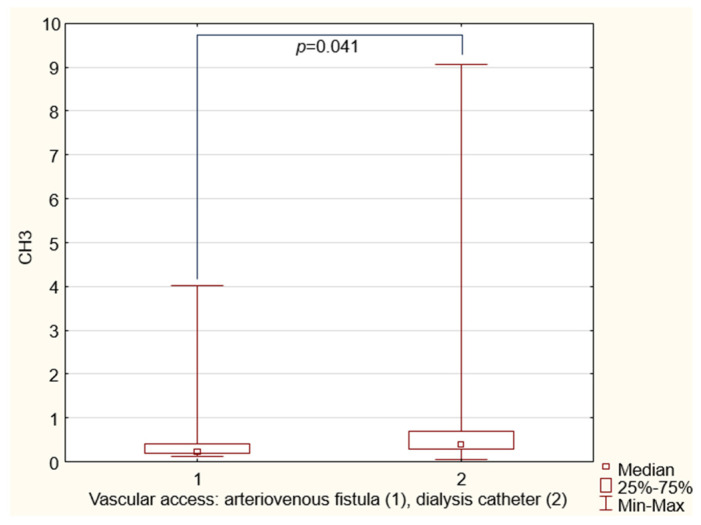
Comparison of CH3 concentrations in patients with a dialysis catheter versus an arteriovenous fistula (*p* = 0.03).

Higher CH3 concentrations were observed in patients with a dialysis catheter versus an arteriovenous fistula (*p* = 0.03). Figure 4. 

**Figure 5:**
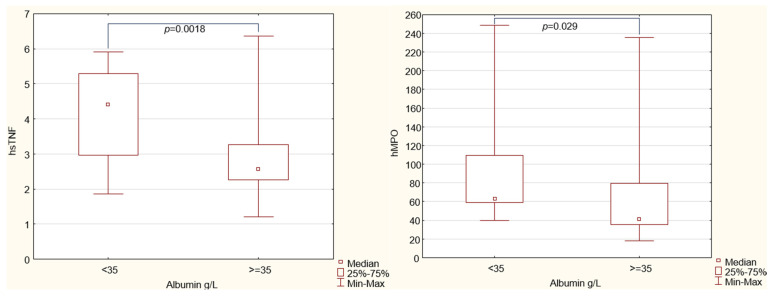
Comparison of hsTNF and hsMPO in groups with albumin concentrations of <35 g/L and ≥35 of TNF (*p* = 0.001) and MPO (*p* = 0.03), respectively.

Patients with albumin levels below the normal range were compared with those within the normal range; higher TNF levels (*p* = 0.001) and MPO levels (*p* = 0.03) were found in the group with lower albumin concentrations. Figure 5.

**Figure 6:**
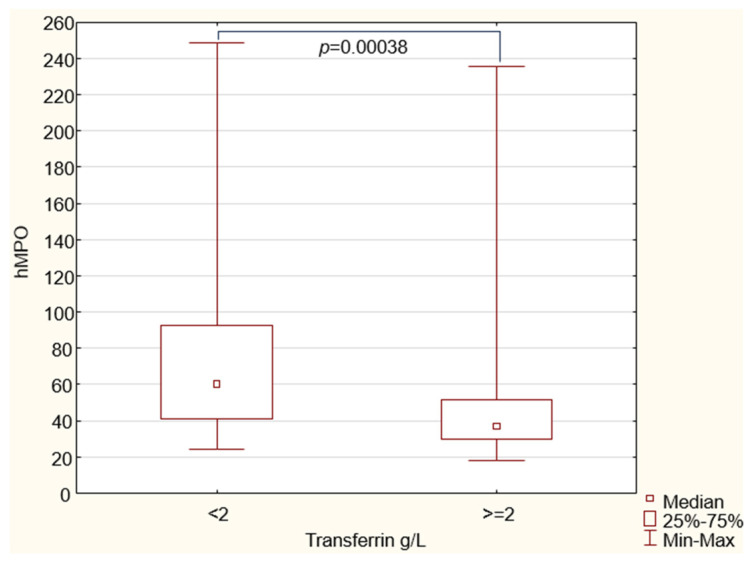
Comparison of MPO concentrations between the group with a low transferrin content of <2 g/L (below normal) and the group with a normal transferrin content of ≥2 g/L (*p* = 0.0004).

Patients with transferrin levels below the normal range were compared with those within the normal range. Higher MPO levels were observed in the group with lower transferrin concentrations (*p* = 0.0004). Figure 6.

#### 2.3.1. Fat-Free Mass Index (FFMI)

Regarding the FFMI, no statistically significant differences were observed between the low, medium, and high tertiles in terms of either inflammatory markers (hs-CRP, IL-6, TNF-α, IL-1β) or nutritional parameters (albumin, transferrin). These results suggest that in this cohort of maintenance hemodialysis patients, variations in lean body mass alone were not a primary driver of systemic inflammation or a differentiator of nutritional status.

#### 2.3.2. Fat Mass Index (FMI)

In contrast, stratification by FMI revealed a significant relationship with systemic inflammation. Specifically, levels of high-sensitivity C-reactive protein (hs-CRP) were significantly higher in the medium and high FMI tertiles compared to the low tertile (*p* = 0.008 and *p* = 0.01, respectively). Interestingly, there was no statistically significant difference in hs-CRP levels between the medium and high FMI tertiles. No significant differences were found across FMI tertiles regarding nutritional parameters.

Age and sex had no effect on inflammation. Age and sex had an impact on body composition: as age increased, the amount of adipose tissue—including visceral fat—increased, while muscle mass decreased; similarly, in men, both the absolute amount and percentage of muscle mass were higher, while those of adipose tissue were lower. There was no difference in the amounts of visceral fat between the two sexes.

### 2.4. Analysis of Deaths During the 12-Month Follow-Up Period

During the 12-month follow-up period, 19 patients died.

When the group of deceased patients was compared with those alive at the end of the 12-month follow-up, the following findings were made for the former of the two:

(1) Higher TNF levels (*p* = 0.0007)
(2) Lower albumin levels (*p* = 0.0004)
(3) A lower phase angle (*p* = 0.04). Cf. Table 5.

In a logistic regression model that also accounted for age and sex, high TNF-α levels (*p* = 0.0123, after logarithmic transformation) and low albumin levels (*p* = 0.0120) were independent predictive markers of death in HD patients over the 12-month follow-up. Cf. Table 6.

## 3. Discussion

(1) BMI does not correlate with any inflammatory markers or nutritional status markers, such as albumin, transferrin, and cholesterol.

Obesity is becoming an increasingly serious problem worldwide [19]. Excess body weight is associated with an increased risk of CKD in the general population [20]. In our study group, BMI proved insufficient for a comprehensive characterization of the participants. No significant correlations were found between BMI and inflammatory markers or nutritional status markers. When measured separately, this parameter proved to be clinically irrelevant and did not affect the significance of the results obtained. This view is supported by the findings of Stenvinkel et al. [21], who noted that raw BMI did not reflect the severity of inflammation. However, when the study group was stratified into normal weight, overweight, and obesity categories, we observed that patients with obesity had significantly higher IL-6 concentrations compared to both normal-weight and overweight individuals. A detailed analysis regarding the significance and implications of IL-6 levels can be found in Section 3 (Figure 1).

In the general population, the risk of death increases as BMI rises. However, in the HD population, factors commonly considered to indicate cardiovascular risk—such as obesity, high cholesterol, and hypertension—paradoxically appear to have a beneficial effect on the patients [21]. One possible explanation for this paradox is the malnutrition-inflammation complex syndrome (MICS), which is common among patients with end-stage renal disease and is the leading cause of cardiovascular mortality in this group, rather than the traditional cardiovascular risk factors. It has also been suggested that this reverse epidemiology may be associated with higher albumin levels, a lower risk of energy deficiency, and higher cholesterol and lipoprotein concentrations in patients with higher body weight [22,23,24].

On the other hand, this phenomenon may be due to the lack of accuracy in the BMI measurement itself. A study by Lin et al. [25] highlighted the superiority of body composition measurements—and in particular body fat percentage—as a predictor of mortality over BMI, the latter failing to account for muscle atrophy, a common occurrence in CKD patients. It may incorrectly classify individuals with sarcopenic obesity as normal weight patients. Therefore, the MIS score proves to be more useful; as an inflammatory marker in HD patients, it is a very good predictor of mortality in this group [26], while low MIS score patients reported a significantly better quality of life [27]. Another useful tool is the bioelectrical impedance method [28].

(2) Body composition significantly impacts hs-CRP and IL-6 levels, with higher adipose tissue and lower muscle mass driving inflammation.

Inflammation is a physiological defense response of the human body [29], but it becomes a pathological condition when it is excessive, uncontrolled, and chronically progressive [30,31]. One indicator of inflammation may be interleukin-6, a key cytokine of the immune system that coordinates inflammatory processes. Its excessive synthesis leads to an increased and generalized inflammatory process, which plays a significant role in CKD [32]. Another important and easily monitored marker is high-sensitivity C-reactive protein (hs-CRP), elevated levels of which have a negative impact on survival in both the general population [33] and among HD patients [34].

Our research showed that body composition significantly influenced the levels of both of the aforementioned inflammatory markers. The results indicated that hs-CRP and IL-6 levels were higher in patients with greater body fat (FMI [kg,%]) and lower in those with greater muscle mass (SMM [kg,%]). As the percentage of body fat increased, so did the levels of hs-CRP and IL-6 in the study group. In contrast, as muscle mass increased, the levels of these inflammatory markers decrease significantly. 

To further refine these observations, we performed a comparative analysis stratified by tertiles of body composition indices. Interestingly, while no statistically significant differences in inflammatory or nutritional parameters were found across the Fat-Free Mass Index (FFMI) tertiles, the Fat Mass Index (FMI) proved to be a significant differentiator for systemic inflammation. Specifically, hs-CRP concentrations were significantly higher in both the medium and high FMI tertiles compared to the low FMI tertile (*p* = 0.008 and *p* = 0.01, respectively). The observation that the medium and high tertiles did not differ significantly suggests a “threshold effect,” where even a moderate increase in fat mass is sufficient to significantly elevate systemic inflammatory activity (Figure 3).

Similar conclusions were drawn in a 2025 single-center study, which found that patients with higher body fat mass had higher levels of hs-CRP [35]. A study by Ishimura et al. [5] conducted on a group of 425 HD patients indicated that high-sensitivity CRP was significantly higher in individuals with more adipose tissue, particularly visceral fat. Furthermore, CRP and IL-6 are good indicators of excessive inflammation, and their levels are higher in patients with a higher BMI, as indicated in the report by Delgado et al. [6].

As for IL-6, our results were consistent with those of Kaizu et al. [8] of 2003, which was one of the first studies to demonstrate a direct link between pro-inflammatory cytokines and muscle loss in HD patients. Patients with higher muscle mass percentages had lower IL-6 and CRP levels. In addition, CRP is independently associated with muscle mass in HD patients, making it an independent risk factor for muscle atrophy, which translates into its higher levels in sarcopenia [9]. This directly confirms a link between sarcopenia and excessive inflammation.

Through the results of our study, we aim to highlight the critical importance of muscle tissue in this patient population. Using a professional medical body composition analyzer, our patients were divided into four subgroups. Patients with increasing sarcopenic obesity versus those with increasing muscle mass had significantly higher levels of CRP (10.5 vs. 3.7, *p* < 0.002) and IL-6 (10.14 vs. 5.8, *p* < 0.029). A similar pattern emerged when comparing patients with increasing obesity to those with increasing muscle mass—with inflammatory markers being higher in the former of the two groups (CRP: 11.4 vs. 3.68 *p* < 0.0035; IL-6: 10 vs. 5.75 *p* < 0.025). These results indicate that muscle tissue plays a protective, anti-inflammatory role in the HD group.

(3) Higher visceral fat levels increase IL-6 concentrations.

With hemodialysis patients, it is important to distinguish between obesity defined as an elevated BMI and visceral obesity, the latter being characterized by an increased amount of visceral fat and occurring even in individuals with a normal BMI. When comparing these two groups, Baberashvili et al. [36] noted that patients with visceral obesity and a normal BMI exhibited a more atherogenic profile in terms of inflammatory markers and adipokine expression versus patients with an elevated BMI [37]. Adipose tissue exhibits pro-inflammatory activity, leading to an increase in chronic inflammation through the production of cytokines [7,38].

In our study, we found that IL-6 was positively correlated with the amount of visceral fat (VAT [l]). This was confirmed by a study by Zhang et al. [39], where a positive correlation between visceral fat mass and cytokine levels were demonstrated in older adults. This topic was further explored by Dilaver et al. in 2025 [40] with their paper focusing work on intramuscular adipose tissue (IMAT). Their team noted that HD patients had higher IMAT levels compared to individuals without CKD, highlighting a positive correlation between IMAT, body composition, and elevated levels of inflammatory markers such as IL-6. The more IMAT, the lower the handgrip strength, which is a good and reliable indicator of muscle function and, consequently, of progressive sarcopenia [41]. Also, elevated IL-6 levels are known to be associated with sarcopenia [42], a finding supported by reports showing that a higher MIS score is positively correlated with elevated IL-6 levels [43]. Istanbuly et al. [44] highlighted the association between this cytokine and all-cause mortality in HD patients. Furthermore, higher levels of this interleukin are also associated with an increased incidence of cardiovascular events in this group due to accelerated progression of coronary artery calcification [45].

(4) NET activation markers do not have a significant effect on body composition. NET formation grows with the duration of HD treatment and in patients with vascular access via a dialysis catheter. High CH3, MPO levels are associated with low albumin and transferrin levels.

In our study, we took an unconventional approach to investigating inflammation in hemodialysis patients. In addition to standard inflammatory markers (hs-CRP, IL-1B, IL-6, TNF-α), the following markers of NET activity were assessed as well: CH3 (citrullinated histones), MPO (myeloperoxidase), and PMN elastase (neutrophil elastase). We were guided by the research on chronic inflammation, neutrophil activation, NETosis, and its complications in hemodialysis patients [11,12,18]. The chronic inflammation that develops in this group has a complex etiology, comprising factors related to both renal replacement therapy and other agents [13,46,47,48,49]. Constant stimulation of the immune system is not without significance in activating neutrophils and the NETs they produce.

Our study pointed to a positive correlation between NET formation and renal dialysis vintage. Bieber et al., in their study of the effects of modern dialysis membranes on the immune system, demonstrated that a single hemodialysis session alone led to neutrophil activation and the release of their extracellular traps [14]. This finding was supported by a 2024 cross-sectional study by Emana R. Edris et al. [15]. It should be noted that both studies indicated a higher baseline level of NET activity markers in CKD patients compared to the control group; however, they highlighted a significant increase in neutrophil activity following a single HD session.

Our study, too, confirmed the finding reported in the literature regarding the effect of the type of vascular access on inflammation, demonstrating a positive correlation between the presence of a dialysis catheter and neutrophil activity (Figure 4). Stuart L. Goldstein et al. found that the mere presence of an uninfected dialysis catheter was responsible for elevated CRP levels. Furthermore, in the group of patients who successfully developed an AVF during the study, there was a significant decrease in CRP levels (by as much as 82%, *p* < 0.0001) and an increase in albumin and hemoglobin levels [16]. Another study by Mihaly B. Tapolyai et al. showed that patients with a dialysis catheter not only had higher CRP levels but also a higher neutrophil-to-lymphocyte ratio (NLR) compared to those with an AVF, which demonstrated that the dialysis catheter kept neutrophils in a state of constant mobilization [50].

Chronic inflammation is accompanied by persistently high levels of pro-inflammatory cytokines, including IL-6 and TNF-α, which are believed to have anorexigenic effects [51,52]. A study by Kamyar Kalantar-Zadeh’s team demonstrated that a lack of appetite in HD patients was one of the simplest and least expensive indicators suggesting severe inflammation [4]. Anorexia, meanwhile, was positively correlated with hypoalbuminemia and higher MIS scores, indicating the patient’s worsening cachexia (MICS) [4]. It was therefore not surprising that our study found a significant negative correlation between CH3, a marker of NETosis, and albumin levels. Furthermore, significantly higher concentrations of myeloperoxidase (MPO)—another key indicator of NETosis—were observed in the group of patients whose albumin and transferrin levels were below the reference range. (Figure 5 and Figure 6). In a study by Yue Zheng et al., albumin demonstrated antioxidant properties against mtROS, thereby inhibiting the NET activation cascade [53]. Albumin at physiological concentrations is believed to act as an inhibitor of extracellular trap release [54,55]. However, one cannot overlook the finding reported in the study by Daria V. Grigorieva et al. regarding the effect of MPO on albumin [55]. This enzyme, released during NETosis, causes the formation of HOCl, a powerful oxidizing agent that also oxidizes albumin. The resultant HOCl-modified HSA become neutrophil activators. When these factors are considered together, a vicious cycle becomes apparent: HD patients suffer from chronic inflammation and malnutrition. The associated hypoalbuminemia correlates with increased NET activity, which, in turn, can modify albumin so that it activates additional neutrophils.

Our study rendered an interesting result regarding the NETosis process and body composition. Patients with increasing obesity, compared to the group with increasing muscle mass, were characterized by significantly higher levels of PMN—an indicator of NETosis (49.74 vs. 35.43, *p* = 0.002). This indicated that muscle tissue played a protective, anti-inflammatory role in the HD group, as described earlier in this paper.

(5) The main predictors of death during a 12-month follow-up in HD patients are albumin and TNF-α levels.

In our study, we demonstrated that lower albumin levels were associated with an increased risk of death during the 12-month follow-up period. An important study in this area is the research conducted by Kamyar Kalantar-Zadeh’s team, which demonstrated how the risk of death increased dramatically as albumin levels declined in HD patients [56]. This study emphasized that it was the dynamic analysis of albumin levels—specifically, the direction of change in albumin concentration (i.e., an increase or decrease)—that helped predict survival. This indicated that, in this group of patients, it was crucial to know not only the current albumin level but also the levels from previous months. Even more importantly, this retrospective cohort study of over 58,000 patients receiving chronic hemodialysis showed that as many as 19% of deaths in this population could potentially be prevented by maintaining albumin levels above 3.8 g/dL. The association between low albumin levels and mortality was confirmed by a study by George A. Kaysen et al., which also explained the physiological mechanisms responsible for the decrease in albumin levels among patients undergoing renal replacement therapy [57]. In addition to the impact of diet, the researchers emphasized the impact that inflammation had on hypoalbuminemia. It was shown that inflammation was responsible for the increased catabolism of these proteins.

According to our findings, TNF-α levels also proved to predict death in a 12-month follow-up period. Le Viet Thang’s team reached a similar conclusion after observing 319 patients undergoing low-flow dialysis over a 3-year period [58]. In the study population, which was divided into three subgroups based on TNF-α levels, the group with the highest concentration of this pro-inflammatory cytokine was associated with significantly higher overall mortality compared to the other two groups. One article worth mentioning in the context of the relationship between TNF-α and mortality is the study by Beata Marie R. Quinto et al. [59], which examined the effects of removing inflammatory factors from the blood in critically ill AKI patients. The main finding of this study was the established association between the filter’s high capacity to remove TNF-α and a better survival rate. Furthermore, a low capacity of the filter to remove TNF-α was found to be an independent risk factor for death, even after adjusting for the patient’s condition. Although this study focused on a population other than patients on chronic dialysis, it highlighted the significant role of tumor necrosis factor-alpha in the progression of a patient’s condition.

(6) Factors affecting albumin levels included those that increased it (percentage of muscle mass, a high phase angle) and those that decreased it (CH3,MPO, IL-6, TNF-α).

As mentioned above, in our study, albumin levels were one of the main predictors of death. Therefore, it appears important to understand the relationships between albumin and other parameters. Our observations indicated that both a higher percentage of muscle mass and a high phase angle increased albumin levels.

The phase angle is determined based on the resistance and reactance values obtained from the bioelectrical impedance analysis. This parameter, which is influenced by the integrity and functionality of cell membranes, has become an indicator of cellular health, a surrogate marker of muscle quality [60,61], a predictor of sarcopenia [62], and a predictor of PEW in patients on chronic dialysis [63]. In this group of patients, an acute (low) phase angle may indicate malnutrition, overhydration, or a combination of both. A sufficiently high phase angle is associated with good nutritional status, greater muscle mass, lower overhydration, and better prognosis. This was reflected in our results, which indicated a correlation between a high phase angle and higher albumin concentrations, and thus better nutritional status, reduced inflammation, and a more favorable prognosis.

In the human body, there are two main categories of protein: somatic protein (skeletal muscle) and visceral protein. In their paper, D. Fouque et al. highlighted the term PEW (protein-energy wasting), using it to replace the concept of malnutrition itself [64]. In patients with ESRD, this is a complex issue. In addition to a loss of appetite, the depletion of protein and energy reserves is also a result of chronic inflammation. Pro-inflammatory cytokines reduce the production in the liver of so-called “negative” acute-phase proteins, including albumin and prealbumin, leading to a decrease in visceral protein levels. It is only as a result of prolonged catabolism that the ubiquitin-proteasome pathways are activated, leading to the catabolism of skeletal muscle. Thus, it can be concluded that patients with a higher percentage of muscle mass—and therefore higher albumin levels—exhibit less severe inflammation.

Therefore, further results of our study indicating that CH3, MPO, IL-6, and TNF-α contributed to a decrease in albumin levels did not come as a surprise. These findings were consistent with the latest ASPEN guidelines (David C. Evans et al.), which emphasize that visceral proteins are primarily negative acute-phase proteins and should not be used in isolation in diagnosing malnutrition, as their serum concentrations correlate more strongly with the intensity of the inflammatory response than with nutritional status itself [65]. This phenomenon can be explained by the liver’s prioritization of inflammatory protein synthesis and plasma protein redistribution resulting from increased vascular permeability during inflammation.

(7) Factors affecting TNF-α levels: inflammatory markers such as elastase, IL-1β, hs-CRP, and IL-6 increase these levels.

TNF-α is an inflammatory cytokine that plays a key role in inflammation, apoptosis, and anticancer defense. In our study, we demonstrated that inflammatory markers such as neutrophil elastase, IL-1β, IL-6, and hs-CRP increased levels of tumor necrosis factor-alpha.

It is worth noting that cytokines are components of a complex signaling network in the body, in which target cells are exposed to various combinations of cytokines that can exert additive, inhibitory, or synergistic effects, as appropriate [66]. The aforementioned markers of inflammation can be considered from a similar standpoint. Contact between blood and the dialysis membrane triggers neutrophil activation and the release of NETs, one of the components of which is neutrophil elastase [45]. A study by Mężyk-Kopeć et al. suggested that proteolytic enzymes—neutrophil elastase and cathepsin G—might release the biologically active form of s-TNF-α circulating in serum through their ability to ‘cleave’ m-TNF-α, initially anchored in the cell membrane [67]. This cascade of inflammation could then lead to an increased expression of IL-1 and IL-6 by TNF-α. A study by Turner et al. identified intracellular signal transduction pathways through which TNF-α induced the expression of IL-1 and IL-6 in human cardiac fibroblasts, contributing to cardiac remodeling [68]. In the context of patients on chronic dialysis, this study illustrates how inflammatory cytokines are responsible for the clinical implications observed in this patient group, such as progressive circulatory failure.

Furthermore, patients undergoing renal replacement therapy showed a significant increase in endogenous inhibitors of pro-inflammatory mediators; paradoxically, these inhibitors were unable to fully neutralize the biological activity of IL-1 and TNF-α, which evidenced immune dysregulation in these patients [69]. Tumor necrosis factor-alpha causes an increase in CRP levels indirectly, via IL-6 [70]. In turn, the CRP leads to an increase in the levels of TNF-α, IL-1beta, and IL-6 [71]. The above illustrates the self-amplifying vicious cycle of inflammation in HD patients: neutrophil elastase, emerging through NETosis, leads to increased levels of TNF-α, which, via IL-6, causes the liver to prioritize the synthesis of inflammatory proteins, which in turn escalate inflammation through a positive feedback loop. Consequently, this leads to the aforementioned MICS.

(8) No association was found between body composition indices and survival during the 12-month follow-up period.

One method of analyzing body composition in adults involves the use of so-called body composition indices, which are calculated using fat mass (FM), fat-free mass (FFM), and total body water (TBW) [72]. They enable metabolic and endocrine characterization, contributing to patient profiling and the identification of risks associated with abnormal values of these indices [73]. The available literature contains ample evidence supporting their high usefulness. One example is a study conducted by Kang et al. [74], which showed that fat-free mass (FFM) was useful in determining bone mineral density (BMD). This was described from another perspective by Baldessari et al. [75], who demonstrated that a comprehensive assessment of the patient, including their nutritional status, could have a positive impact on further treatment. For this reason, there are serious grounds for a widespread use of indices such as FM, FMI, FFM, and TBW, as they help to account for the highly variable body composition despite a similar interpretation of the body mass index (BMI) in different patients [76].

This topic is widely discussed nowadays, particularly with regard to patient mortality. Overweight and obesity are well-known to be significant and key risk factors for cardiovascular disease (CVD) [77], which is associated with a higher risk of death from the disease [78]. In contrast, however, the term “obesity paradox” has emerged to describe an aspect that manifests itself repeatedly in certain medical conditions as based on the protective association between high BMI values and mortality [79]. Nevertheless, there is a growing recognition of the limitations and shortcomings of this method as a measure of obesity (BMI), with suggestions that the assessment of body fat and fat-free mass—the latter of which has often been overlooked—is crucial for the overall results of the tests [80]. One example is a study conducted by Graf et al. [81], which led to the conclusion that a decrease in the fat-free mass index (FFMI) was associated with increased mortality among older adults.

Our study, in turn, allowed us to assess the relationship between body composition indices and their impact on HD patient survival. Based on the results obtained, it can be concluded that the indicators in question do not have a direct impact on the survival of patients undergoing HD during the 12-month follow-up period. The main predictors of death in the study patients are albumin and TNF-α levels (cf. Point 6 of the conclusions). It is worth noting that the narrow time frame available for our study significantly limited our ability to conduct a broader analysis of the impact of body composition indices on patient survival. The above must lead to the obvious conclusion that continuing the research over the long term could yield interesting results—which may differ significantly from the conclusion reached so far.

(9) No association was found between NET markers and survival during the 12-month follow-up period.

NETosis is described as the process of forming a specialized network composed of DNA and antimicrobial proteins released by activated neutrophils, which helps neutralize pathogens but also has pro-inflammatory and pro-thrombotic potential [82]. Hemodialysis, which is used to treat chronic kidney disease (CKD), is one of the factors contributing to exacerbation of inflammation in the patients. This occurs through the activation of the innate immune system, particularly the complement system, as well as through the recruitment of neutrophils [83]. It is because of them that the NETosis markers described earlier- such as CH3-citrullinated histones, MPO (myeloperoxidase), and PMN elastase—are elevated in patients undergoing chronic hemodialysis [12].

NET indices, as described in the available literature—particularly during dialysis sessions—have been identified as predictors of poorer treatment outcomes [84]. In addition, a link is increasingly being reported between an exacerbated NETosis and the pathogenesis of many diseases, including sepsis, systemic lupus erythematosus, rheumatoid arthritis, and small-vessel vasculitis [85]. A study by Delabranche et al. [86] demonstrated that NETosis was significantly correlated with disseminated intravascular coagulation (DIC) caused by septic shock. This is an important observation regarding patients on chronic hemodialysis who, as a result of treatment for their underlying condition (CKD), are at risk of developing chronic inflammation manifested by elevated levels of C-reactive protein (CRP) or endothelial dysfunction [87]. These components contribute to the multifaceted activation of NETs [88].

The regulation of NET processes remains the focus of research exploring inhibitory and stimulatory mechanisms [82]. The available literature emphasizes the importance and relevance of research on NETosis and its markers in order to better understand the process that is a key host defense mechanism [89]. Hence, an important conclusion drawn from our study is that we found no association between NET markers and survival in HD patients; however, we did find a significant correlation between NET markers and muscle mass and body composition (Cf. Points 4 and 5). It is important to note the relatively short 12-month follow-up period for patients undergoing HD in this study. Perhaps a long-term observation would be more beneficial.

## 4. Patients and Methods

This study was approved by the Bioethical Committee of the Pomeranian Medical University, Szczecin, Poland (KB-006/17/2025).

### 4.1. Study Group

The study included 99 patients with stage 5 chronic kidney disease (CKD) undergoing maintenance hemodialysis at the University Clinical Hospital No. 2, Pomeranian Medical University, Szczecin, Poland. Patients received standard hemodialysis treatments three times a week, with each session lasting approximately 4 h. The procedures were performed using polysulphone membrane. The standard blood flow rate (Qb) ranged from [250–350 mL/min], and the dialysate flow rate (Qd) was maintained at [500 mL/min]. Anticoagulation was achieved using unfractionated heparin, adjusted individually based on the patients’ clinical needs. At baseline, vascular access types were: arteriovenous fistula (AVF) n = 29 (30%), tunneled dialysis catheter n = 70 (70%). Comparisons of inflammatory and NET markers by access type were assessed. After the patients were enrolled in the study, they were followed for 12 months, during which overall mortality in their group was monitored. All the patients gave their informed written consent to participate in the study. The study consisted of three components: blood sampling to assess inflammatory and nutritional status markers, body composition analysis, and a 12-month follow-up with an analysis of all-cause and cardiovascular mortality.

### 4.2. Body Composition Analysis

The measurement was performed using a professional medical body composition analyzer, the Seca mBCA 525, in accordance with its user manual [90]. The participants’ body weight and height were measured manually prior to the analysis. A brief description of the main body composition parameters measured using the Seca mBCA 252 is provided below (Table 7).

The examination was quick and non-invasive, based on an 8-point measurement of bioelectrical impedance on the patient’s body surface. The electrical current applied was 100 μA; therefore, patients with cardiac devices were excluded from the body composition analysis. Each person was evaluated following their hemodialysis session. The analyzer assigned each person to one of four subgroups based on their body composition chart: increasing sarcopenic obesity, increasing obesity, increasing thinness or increasing muscle mass [6]. The authors also divided the participants into four groups based on their BMI: underweight (<18.5), normal weight (18.5–24.9), overweight (25–29.9), or obese (30 or higher).

### 4.3. Inflammation

Blood was sampled prior to HD treatment into 4 mL EDTA tubes (on the day of the monthly follow-up tests, once at study start). The blood samples were centrifuged at 4000 rpm for 10 min, and the plasma was stored at −80 °C prior to analysis.

The plasma concentrations of classical inflammatory markers hs CRP, hs IL-6, hs IL-1β, and hs TNF-α, as well as markers of NETosis, including citrullinated histone H3, PMN-elastase, and myeloperoxidase (MPO), were measured using commercially available enzyme-linked immunosorbent assay (ELISA) kits. Quantikine HS ELISA kits (Bio-Techne R&D Systems, Abingdon, UK) were used for hs IL-6, hs IL-1β, and hs TNF-α. determinations. A Quantikine ELISA kit (Bio-Techne R&D Systems, Abingdon, UK) was used for the measurement of myeloperoxidase contents. Hs CRP concentrations were determined using ELISA kits from DRG Instruments GmbH (Marburg, Germany). Citrullinated histone H3 was measured using kits from Cayman Chemical (Ann Arbor, MI, USA), and PMN-elastase using kits from Invitrogen, Thermo Fisher Scientific (Waltham, MA, USA). All analyses were performed according to the manufacturers’ protocols. Absorbance measurements were carried out using the BioTek Cytation 5 Cell Imaging Multimode Reader (BioTek Instruments, Winooski, VT, USA).

The concentrations of nutritional status markers albumin and transferrin were determined through routine biochemical tests performed in the Central Laboratory of the University Clinical Hospital No. 2, the Pomeranian Medical University, Szczecin, Poland.

### 4.4. 12-Month Follow-Up

The study group was followed-up for one year, and the overall mortality was assessed.

The analysis made use of patient data such as age, sex, dialysis vintage in months, type of vascular access (catheter, arteriovenous fistula (AVF)), dialysis quality (Kt/V index), and co-morbidities, including cardiovascular disease, cancer, diabetes mellitus, COPD, and autoimmune diseases. The analysis also utilized standard laboratory parameters such as complete blood count, ferritin levels, calcium levels, phosphorus levels, and parathyroid hormone (PTH) levels.

### 4.5. Statistical Analysis

The Shapiro–Wilk test was used to study the normality of distributions of quantitative variables. Since most of the distributions were significantly different from normal (*p* < 0.05), we used nonparametric Mann–Whitney U-test to compare groups and correlations were studied by means of Spearman’s rank correlation coefficient. Data were described as median (lower quartile (25%)–upper quartile (75%)) or mean ± standard deviation. *p*-values were considered significant when <0.05 without correction for multiple testing. To identify independent predictors of 12-month mortality we fitted a multivariable logistic regression model. Candidate predictors were selected based on clinical relevance and significant results of univariable analyses. Statistical analysis was performed using Statistica 13 software (StatSoft, Tulsa, OK, USA).

## 5. Conclusions

In summary, the results of our study paint a coherent and complex picture of the hemodialysis patient. This is a patient in whom obesity (particularly visceral and sarcopenic obesity) drives inflammation. This inflammation is further exacerbated by the dialysis procedure itself, the presence of catheters, and the activation of neutrophils (NETosis). The resulting “cytokine storm” (TNF-α, IL-6, elastase) leads to albumin breakdown, vascular damage, and systemic deterioration, ultimately resulting in death. The observed associations between body composition and inflammatory markers may be bidirectional. Adipose tissue—particularly visceral adiposity—secretes pro-inflammatory adipokines (e.g., IL-6, TNF-α) that can elevate systemic inflammation and suppress hepatic synthesis of visceral proteins, potentially contributing to muscle loss. Conversely, systemic inflammation can drive muscle catabolism and lower lean mass. Therefore, while our cross-sectional analyses demonstrate strong correlations, they do not prove causality. Prospective and interventional studies are required to define causal pathways and temporal sequence. In this context, preserving muscle mass, combating abdominal obesity, avoiding the use of dialysis catheters, and working to reduce inflammation emerge as the primary therapeutic goals.

Strengths: The study group was assessed not only for the presence of typical inflammatory markers, but also for neutrophil activation and NET formation. A detailed body composition analysis was performed using bioelectrical impedance.

Limitations: This was a single-center study. The follow-up to assess overall mortality lasted only 12 months. The study cohort consists of prevalent hemodialysis patients, which may be characterized by a higher baseline inflammatory burden, longer dialysis vintage and increased comorbidity relative to incident populations. This may limit generalizability and could bias associations between inflammation, body composition and outcomes. We explicitly state this as a limitation and recommend validation in incident dialysis cohorts and with longer follow-up.

## Figures and Tables

**Table 1:** Group characteristics.

Overall Participants	Median [25th–75th Percentile]
Age	65 [47–73]
BMI [kg/m^2^]	26.89 [23.7–32.17]
SMM [%]	30 [0.24–0.36]
SMM [kg]	24.8 [19.1–29]
FMI [%]	31.66 [13.7–35.33]
FMI [kg]	22.79 [22.18]
FMI [kg/m^2^]	8.25 [4.5–13.2]
FFM [%]	69.31 [59.5–82]
FFM [kg]	54.15 [48.1–63.5]
FFMI [kg/m^2^]	19.25 [17.1–20.9]
VAT [l]	2.35 [1.2–4.5]
TBW [%]	51.75 [44–59.9]
Phase angle ϕ [◦]	4.9 [4.2–5.5]
Dialysis vintage [months]	36.5 [15–72]
Albumin [g/dL]	41 [38–43]
hs TNF [pg/mL]	2.68 [2.26–3.35]
hs IL-6 [pg/mL]	5.61 [3.14–12.29]
hs-CRP [mg/dL]	5.4 [2.7–12.3]
CH_3_ [ng/mL]	0.37 [0.22–0.68]
PMN [ng/mL]	39.64 [32.43–50.13]
hMPO [ng/mL]	43.21 [36.1–85]
hs IL-1B [pg/mL]	0.27 [0.16–0.38]

Abbreviations: BMI [kg/m^2^]—body mass index; SMM [%]—skeletal muscle mass percentage; SMM [kg]—skeletal muscle mass; FMI [%]—fat mass percentage; FMI [kg]—fat mass; FFM [%]—fat-free mass percentage; FFM [kg]—fat-free mass; FFMI [kg/m^2^]—fat-free mass index; VAT [l]—visceral vat; TBW [%]—total body water; hs TNF—high-sensitivity Tumor Necrosis Factor; hs IL-6—high-sensitivity interleukin-6; hs-CRP—high-sensitivity C-reactive protein; CH3—citrullinated histone; PMN—polymorphonuclear elastase; hMPO—myeloperoxidase; hs IL-1B—high-sensitivity interleukin-1 beta.

**Table 2:** The four groups according to BMI.

BMI Category	BMI Range (kg/m^2^)	Number of Patients (*n*)	Percentage (%)
Underweight	<18.5	2	2.0%
Normal weight	18.5–24.9	35	35.4%
Overweight	25.0–29.9	30	30.3%
Obesity	≥30.0	32	32.3%
Total		99	100.0%

**Table 3:** The nutritional status groups according to body composition.

Nutritional Status (Body Composition)	Number of Patients (*n*)	Percentage (%)
Increasing sarcopenic obesity	*20*	22.2%
Increasing obesity	29	32.2%
Increasing thinness	22	24.4%
Increasing muscle mass	19	21.1%
Total	90	100.0%

**Table 4:** Distribution of albumin and transferrin levels categorized as normal or below the reference range.

Albumin:		
Albumin Level	Number of Patients (*n*)	Percentage (%)
Below the normal range < 35 g/L	10	10.1%
Within the normal range ≥ 35 g/L	89	89.9%
Total	99	100.0%
**Transferrin:**		
**Transferrin Level**	**Number of Patients (*n*)**	**Percentage (%)**
Below the normal range < 2 g/L	60	60.6%
Within the normal range ≥ 2 g/L	39	39.4%
Total	99	100.0%

**Table 5:** Group characteristics by clinical outcome.

Variable	Median [Q_1_–Q_3_]		*p*
	Death = 0	Death = 1	
Age	65 [48–72.5]	67 [45–73]	0.880
Dialysis vintage [months]	36 [15–72]	37 [18–82]	0.693
Albumin [g/dL]	41 [39–43]	37 [33–40]	0.0004
hs TNF [pg/mL]	2.55 [2.126–3.197]	3.70 [2.711–4.876]	0.0007
hs Il-6 [pg/mL]	4.8 [3.116–10.804]	10.97 [4.584–16.616]	0.064
hsCRP [mg/dL]	5.1 [2.7–9.9]	8.4 [1.5–23.1]	0.229
CH3 [ng/mL]	0.37 [0.19–0.71]	0.4 [0.31–0.68]	0.366
PMN [ng/mL]	37.66 [32–50.59]	41.85 [37.88–49.68]	0.509
hMPO [ng/mL]	42.01 [34.372–84.999]	60.6 [36.715–86.268]	0.462
hs Il-1B [pg/mL]	4.8 [0.152–0.382]	0.33 [4.584–16.616]	0.336
Phase angle ϕ [◦]	5 [4.4–5.5]	4.2 [3.4–4.9]	0.041
SMM [%]	30 [24.9–35.71]	28.8 [26.3–38.31]	0.532
SMM [kg]	24.9 [19–29]	23 [21.3–27.2]	0.963
FMI [%]	32.03 [18.94–40.7]	24.47 [12.86–36.26]	0.399
FMI [kg]	23.08 [13.69–35.35]	15.01 [8–32.06]	0.344
FFM [%]	69.25 [60.17–79.76]	77.53 [53.69–85.73]	0.547
FFM [kg]	54.35 [48.13–63.49]	53.53 [49.45–63.17]	0.904
FFMI [kg/m^2^]	19.2 [17.1–20.8]	19.7 [17.4–21]	0.818
VAT [l]	2.3 [1.3–4.5]	2.9 [0.6–4.5]	0.477
BMI	26.67 [23.734–32.232]	26.89 [23.671–31.347]	0.957

Abbreviations: hs TNF—high-sensitivity Tumor Necrosis Factor; hs IL-6—high-sensitivity interleukin-6; hs-CRP—high-sensitivity C-reactive protein; CH3—citrullinated histone; PMN—polymorphonuclear elastase; hMPO—myeloperoxidase; hs IL-1B—high-sensitivity interleukin-1 beta; SMM [%]—skeletal muscle mass percentage, SMM [kg]—skeletal muscle mass; FMI [%]—fat mass percentage; FMI [kg]—fat mass; FFM [%]—fat-free mass percentage; FFM [kg]—fat-free mass; FFMI [kg/m^2^]—fat-free mass index; VAT [l]—visceral fat; BMI—body mass index.

**Table 6:** Multivariable logistic regression model predicting patient death during 12 months of observation.

Independent Variables	OR	−95%CI	+95%CI	*p*
Male sex	0.304	0.064	1.435	0.1262
Age [years]	0.992	0.947	1.039	0.7255
Serum albumin [g/L]	0.816	0.694	0.959	0.0120
Log (serum hsTNF [pg/mL])	17.006	1.784	162.073	0.0123

**Table 7:** The main body composition parameters measured using the Seca mBCA 252.

Parameter	Description
BMI—Body Mass Index [kg/m^2^]	A value calculated by dividing body weight by the square of height, traditionally used to classify patients as underweight, normal weight, overweight, or obese.
FFM—fat-free mass [kg], relative to weight [%]	Calculated by subtracting adipose tissue weight from total body weight; can also be expressed as a percentage of total body weight
FFM_kg—fat-free mass [kg]	Indicates the amount of fat-free mass in kilograms
FFMI—fat-free mass index [kg/m^2^]	Describes the amount of fat-free mass relative to height and weight. Similar to BMI.
SMM_kg—skeletal muscle mass [kg]	Describes skeletal muscle mass in kilograms
SMM_%—percentage of muscle mass in body weight [%]	Describes the percentage of skeletal muscle in total body weight
FM—fat mass [kg], relative to weight [%]	Total body fat; the percentage of total body weight that is fat.
FMI—fat mass index [kg/m^2^]	Describes the amount of the adipose tissue relative to height and weight. Similar to BMI.
FMI_%—fat mass [%]	Describes the percentage of the adipose tissue in total body weight
TBW—total body water [L], relative to weight [%]	Total amount of intracellular and extracellular water; approximately 60% of body weight in a person with normal blood volume.
Phase angle φ [°]	Calculated as the reactance to resistance ratio during bioelectrical impedance measurement. Treated as an indicator of cell wall stability. Useful for assessing health risks.
VAT—visceral adipose tissue [L]	Also known as visceral fat; refers to the fatty tissue that surrounds the organs in the abdominal cavity. Excessive accumulation of visceral fat in the abdominal cavity is known as visceral obesity.

## Data Availability

The raw data supporting the conclusions of this article will be made available by the authors upon request.
